# Radiation-induced alterations of histone post-translational modification levels in lymphoblastoid cell lines

**DOI:** 10.1186/1748-717X-9-15

**Published:** 2014-01-09

**Authors:** Belinda Maroschik, Anne Gürtler, Anne Krämer, Ute Rößler, Maria Gomolka, Sabine Hornhardt, Simone Mörtl, Anna A Friedl

**Affiliations:** 1 Department of Radiation Oncology, Ludwig-Maximilians-University, Munich, Germany; 2 Department Radiation Protection and Health, Federal Office for Radiation Protection, Neuherberg, Germany; 3 Institute of Radiation Biology, Helmholtz Zentrum München, German Research Center for Environment and Health, Neuherberg, Germany; 4 Clinical Cooperation Group “Personalized Radiotherapy in Head and Neck Cancer”, Research Unit of Radiation Cytogenetics, Helmholtz Zentrum München, German Research Center for Environment and Health, Neuherberg, Germany

**Keywords:** Histone modification, Chromatin, Individual radiosensitivity

## Abstract

**Background:**

Radiation-induced alterations in posttranslational histone modifications (PTMs) may affect the cellular response to radiation damage in the DNA. If not reverted appropriately, altered PTM patterns may cause long-term alterations in gene expression regulation and thus lead to cancer. It is therefore important to characterize radiation-induced alterations in PTM patterns and the factors affecting them.

**Methods:**

A lymphoblastoid cell line established from a normal donor was used to screen for alterations in methylation levels at H3K4, H3K9, H3K27, and H4K20, as well as acetylation at H3K9, H3K56, H4K5, and H4K16, by quantitative Western Blot analysis at 15 min, 1 h and 24 h after irradiation with 2 Gy and 10 Gy. The variability of alterations in acetylation marks was in addition investigated in a panel of lymphoblastoid cell lines with differing radiosensitivity established from lung cancer patients.

**Results:**

The screening procedure demonstrated consistent hypomethylation at H3K4me3 and hypoacetylation at all acetylation marks tested. In the panel of lymphoblastoid cell lines, however, a high degree of inter-individual variability became apparent. Radiosensitive cell lines showed more pronounced and longer lasting H4K16 hypoacetylation than radioresistant lines, which correlates with higher levels of residual γ-H2AX foci after 24 h.

**Conclusion:**

So far, the factors affecting extent and duration of radiation-induced histone alterations are poorly defined. The present work hints at a high degree of inter-individual variability and a potential correlation of DNA damage repair capacity and alterations in PTM levels.

## Background

It is becoming increasingly evident that the cellular response towards DNA damage is affected by the structure of the chromatin region surrounding the damage site [1], while at the same time the chromatin structure is affected by the damage response [2]. DNA double-strand breaks (DSBs) elicit a response in an Mbp-large chromatin region surrounding the break that involves alterations in several post-translational modifications (PTMs). Phosphorylation of histone variant H2AX at serine 139 (S139) to yield γ-H2AX is a hallmark step in the cellular response to DSB. The γ-H2AX chromatin domains, which can be visualized as ionizing radiation induced foci (IRIF), delineate regions where a large variety of signalling and repair proteins accumulate [3].

Immunofluorescence detection of PTMs demonstrated alterations in several modifications in the γ-H2AX domain following DSB induction that are associated with regulation of chromatin accessibility, recruitment of DNA damage response factors, and regulation of DNA metabolism and transcription [4]. In some instances, the PTM alterations can also be detected on a more global, i.e. nucleus-wide, manner, e.g. by Western Blot analysis or by analysis of pan-nuclear immunofluorescence staining. Tjeertes et al. [5] conducted in U2OS cells a screen for PTMs that alter both after 24 h incubation with hydroxyurea (a drug that inhibits replication by decreasing the production of desoxyribonucleotides) and 2 h incubation with phleomycin (a drug that induces strand breaks). Only PTMs that exhibited a drastic intensity change in Western Blot-based analysis were considered further, thus raising the possibility that DSB-specific PTMs or PTMs that exhibit only a small alteration were neglected. We decided to conduct a screen to identify PTMs that specifically alter in response to irradiation. In our screen, we used an immortalized normal human lymphoblastoid cell line (LCL). Cells were irradiated with different doses (0 Gy, 2 Gy, 10 Gy) and incubated for 15 min, 1 h and 24 h. Quantitative Western Blot analysis was performed in order to ascertain detection of small alterations. We here report that histone methylation marks exhibit little alteration, except for tri-methylation of H3K4 the levels of which were consistently reduced after irradiation. All acetylation marks tested exhibited a rather long-lasting, globally detectable hypoacetylation after irradiation. Histone acetylation marks were also investigated in a panel of LCLs established from lung cancer patients, where we observed a high degree of inter-individual variability. Long-term hypoacetylation of H4K16 was strongest in cell lines exhibiting increased radiosensitivity and enhanced levels of residual γ-H2AX foci.

## Materials and methods

### Tissue culture and irradiation

Screening experiments were conducted with an Epstein-Barr virus (EBV)-immortalised LCL of a healthy male donor (HuKo). The cells were cultivated in RPMI medium in 75 cm^2^ flasks (37°C, 5% CO_2_) supplemented with 10% FCS and 1% penicillin/streptomycin. Irradiation with 2 Gy or 10 Gy was performed with a ^137^Cs-source (HMW-2000, Markdorf, Germany; dose rate: 0.54 Gy/min) at room temperature. Further experiments were conducted with EBV-immortalised LCLs established from young cancer patients of the LUCY study (LUng Cancer in the Young, http://www.helmholtz-muenchen/epi/) that differ in radiosensitivity, as tested with Trypan Blue and WST-1 assays (Gürtler et al. 2010). Since the nomenclature of cell lines differs between Gürtler et al. 2010 and the present work, Table 1 lists the names and relative radiosensitivity of the lines used. Line 4008, which was not described in the previous study by Gürtler et al. 2010, exhibits survival characteristics comparable to the sensitive line 36011 (data not shown). All investigations on these cell lines were approved by the Ethics Committee of Bavaria (Germany). An LCL established from an Ataxia teleangiectasia patient (Coriell Institute, NJ, USA: GM03189) and an LCL from the patient’s brother with functional ATM (Coriell Institute: GM03323) were used for comparison.

**Table 1:** Cell lines from LUCY cohort and controls

**Name in present work**	**Name in Gürtler et al. 2010**	**Radiosensitivity status**
ATM +/+	2+	Resistant
20037	3	Resistant
4064	9	Resistant
4008	n.d.	Sensitive
4028	14	Sensitive
36011	6	Sensitive
4060	17	Sensitive
ATM −/−	1-	Sensitive

### Preparation of protein extracts

Cells were incubated after irradiation for 15 min, 1 h or 24 h at 37°C, 5% CO_2_ and then collected by centrifugation (5 min, 800 rpm). After washing the cells twice with ice cold PBS the proteins were extracted with RIPA-buffer (150 mM NaCl, 1% NP-40, 10 mM MDOC, 0.1% SDS, 50 mM Tris pH 8.0) on ice. After denaturation for 10 min at 100°C the DNA was removed after one freeze-and-thaw cycle by centrifugation (90 sec, 15000 rpm).

### Immunoblotting and quantitative Western analysis

The proteins were separated with Laemmli loading dye on 10% Bis-Tris NuPAGE-gels (Invitrogen) or 12% TGX-Precast Gels (BioRad). After immunoblotting, membranes were cut in halves, blocked with Roti-Block (Roth) or milk (depending on the antibody (see Table 2)) and incubated with primary antibodies in the indicated blocking solution before detection with appropriate secondary antibodies m-HRP and r-HRP (Santa Cruz; sc2004 and sc2005, respectively). Blots were developed with Amersham ACL Advance (GE Healthcare). Chemiluminescence was detected and images were acquired with a CHEMISMART documentation system (Peqlab, Vilber Lourmat) and the Chemi-Capt 5000 software. Quantitative analysis was performed with the Bio-1D software (Vilber Lourmat). In order to ascertain detection of small differences between samples, blots were only evaluated when in all lanes to be compared directly equal amounts of protein extracts had been loaded. This was determined by equal intensity of the Tubulin alpha signals (max. 20% variation over all lanes to be compared). The signals were normalized with respect to the unirradiated samples at each time point and the Tubulin alpha signals. Data are from at least 2 independent experiments. Significance of deviation from controls was determined by t-tests.

**Table 2:** Antibodies used in this study

**Antibody**	**Company**	**Order nr.**	**Dilution**	**Blocking solution**
γ-H2AX	millipore	05-636	1:1000	Roti-Block
H3K4 unmodified	millipore	05-1341	1:1000	Roti-Block
H3K4me1	abcam	ab8895	1:1000	Roti-Block
H3K4me2	abcam	ab32356	1:1000	Roti-Block
H3K4me3	abcam	ab12209	1:1000	Roti-Block
H3K9ac	millipore	06-942	1:1000	Roti-Block
H3K9me1	millipore	07-450	1:1000	Roti-Block
H3K9me2	millipore	04-768	1:1000	Roti-Block
H3K9me3	millipore	07-442	1:1000	Roti-Block
H3K27me3	millipore	07-449	1:4000	Roti-Block
H3K56ac	millipore	07-677	1:1000	Roti-Block
H4K5ac	upstate	06-759	1:1000	Roti-Block
H4K16ac	millipore	07-329	1:4000	5% milk
H4K20me1	millipore	07-1570	1:1000	Roti-Block
H4K20me2	millipore	07-367	1:1000	Roti-Block
H4K20me3	abcam	ab9053	1:1000	Roti-Block

All antibodies were tested for specificity by peptide competition assays, except for H3K56ac, for which no competing peptide was commercially available. We could exclude, however, that our antibody directed against H3K56ac recognizes H3K9ac, which has a similar target site (Drogaris et al. 2012). All the tested antibodies were specific except for the antibodies detecting the different H4K20 methylation levels, which exhibited some cross-reactivity against the different methylation states.

### Quantitative analysis of γ-H2AX foci

DNA damage-induced phosphorylation of the histone H2AX variant was analyzed 24 h after irradiation with 10 Gy gamma rays. Prior to fixation, cells were spun onto slides for 5 min at 500 rpm using a Cytospin centrifuge. Cells were fixed in 2% PFA/PBS for 15 min, permeabilised in 0.25% Triton X-100/PBS for 5 min, washed three times for 5 min each in PBS and blocked with 1% BSA/PBS three times for 10 min each. Cells were covered with 75 μl of the primary antibody (anti-phospho-Histone H2A.X(Ser139), #05-636, Millipore) diluted 1:200 in 1% BSA/PBS and incubated in a humid chamber at 4°C over night. After washing with PBS (5 min), 0,25% Triton/PBS (10 min), PBS (5 min) and 1% BSA/PBS (7 min), cells were incubated with 75 μl of the secondary antibody (Alexa Fluor 555 goat anti mouse; A-21422, Life Technologies) diluted 1:1000 in 1% BSA/PBS for 45 min at 4°C. Again, cells were washed in 0.25% Triton/PBS two times for 5 min and in PBS two times for 10 min. For counterstaining, cells were incubated with Hoechst-33342 (Sigma-Aldrich) for 2 min and washed in PBS two times. Prior to microscopic analysis, cells were covered with 16 μl Vectashield (Vector Laboratories) and sealed with a coverslip. For foci analysis, an automated scanning and analysis system (Axioplan 2; Carl Zeiss Jena; Metafer4, MetaSystems) was used [6].

## Results and discussion

We screened for alterations in histone modifications detectable by Western Blot analysis of nuclear extracts from a normal LCL (HuKo) after irradiation with two different doses (2 Gy, 10 Gy) and post-irradiation incubation for three different time periods (15 min, 1 h, 24 h). The amount of several of the PTMs tested is known to locally alter within the γ-H2AX domain in response to irradiation. Estimating conservatively that 10 Gy of γ-irradiation induce about 400 DSB, each resulting in a γ-H2AX domain of about 2 Mbp [7], a complete loss of a given PTM within these domains would result in a reduction of the total nuclear signal by about 13% (assuming homogeneous distribution of the PTM within the 6 × 10^9^ bp of the genome). We assume that alterations detectable after 10 Gy may be explained by local events, although more global events cannot be excluded. Alterations detectable after 2 Gy, however, most probably involve some global response reactions.

**Figure 1:**
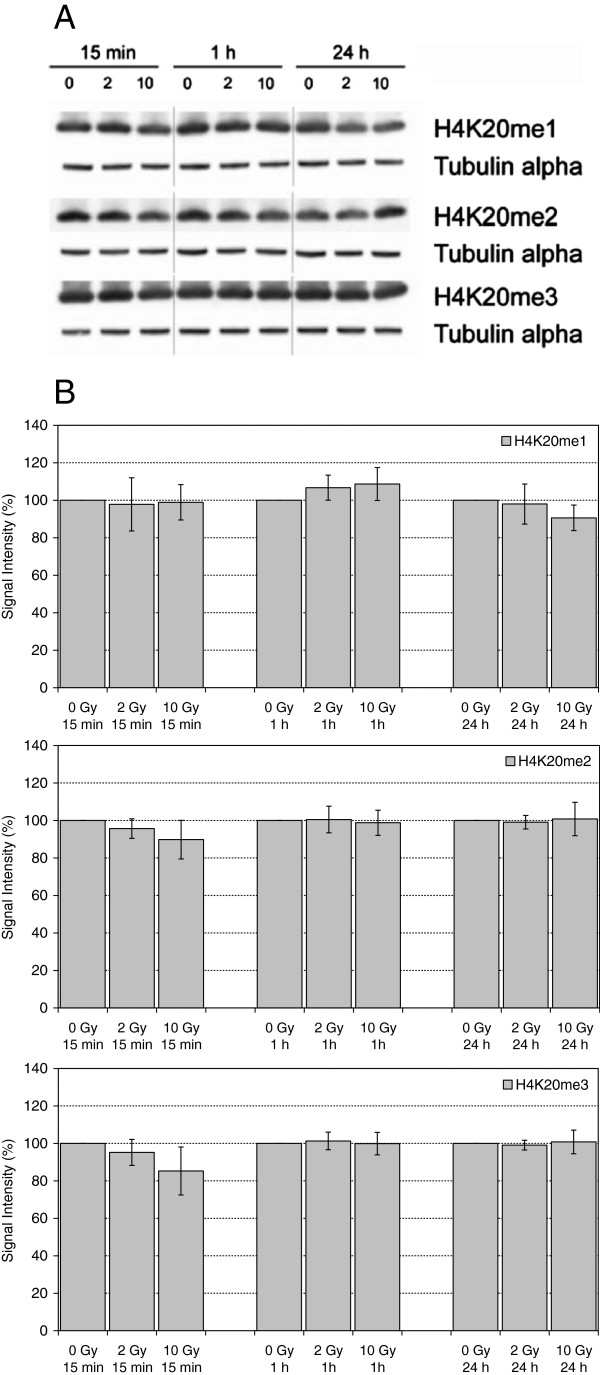
**Levels of H4K20me1, H4K20me2 and H4K20me3 in extracts of HuKo LCLs after irradiation with 0 Gy, 2 Gy and 10 Gy and incubation for 15 min, 1 h and 24 h. A)** representative Western Blot, Tubulin alpha served as loading control. **B)** Quantitative evaluation, each normalized to unirradiated controls. Indicated are mean and standard error of the mean from 2–3 independent experiments and 1–2 blots per experiment.

H4K20 methylation is involved in chromatin compaction and DNA damage response (recent review by [8]). While H4K20me3 is a marker for silenced heterochromatin, H4K20me2 (which is present on 80% of all chromatin-bound H4 molecules in proliferating cells) and H4K20me1 are broadly distributed. H4K20me1 and H4K20me2 play important roles in the DNA damage response as binding sites for 53BP1 [9-14]. Nevertheless, bulk levels of H4K20me1 and H4K20me2, as well as H4K20me3, appear to be quite stable after damage induction [9,11,15,16], although others reported increased mono- and di-methylation [17]. In our study, no significant alterations were detectable in the nuclear amount of H4K20me1, H4K20me2 or H4K20me3 (Figure 1).

**Figure 2:**
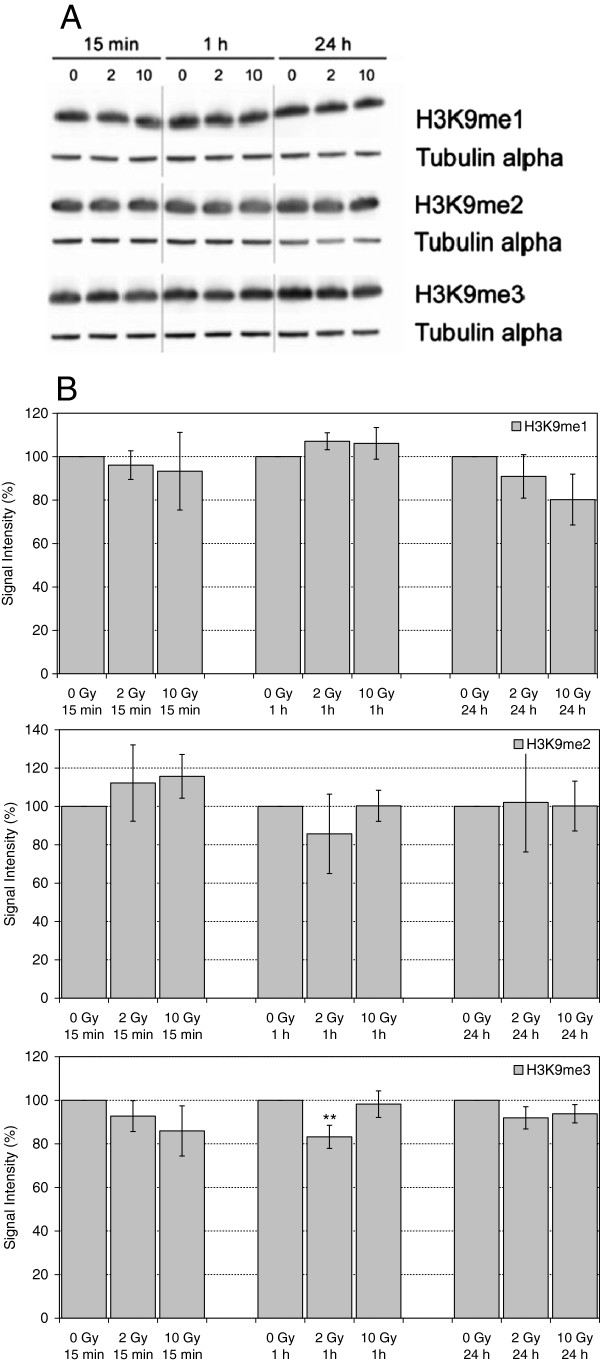
**Levels of H3K9me1, H3K9me2 and H3K9me3 in extracts of HuKo LCLs after irradiation with 0 Gy, 2 Gy and 10 Gy and incubation for 15 min, 1 h and 24 h. A)** representative Western Blot, Tubulin alpha served as loading control. **B)** Quantitative evaluation, each normalized to unirradiated controls. Indicated are mean and standard error of the mean from 2–3 independent experiments and 1–3 blots per experiment. ** statistically significant with 0.005 > α > 0.0005.

H3K9 methylation is involved in transcriptional silencing, formation of heterochromatin regions and DNA methylation. Although H3K9me3 has been implicated in the DNA damage response as a binding site for the histone acetyltransferase TIP60 [18], in many studies alterations in the amount of H3K9me3 or H3K9me2 could not be detected, neither within the γ-H2AX domain nor on a global level ([15,18-23]). Others, however, observed a transient reduction in nuclear H3K9me3 and H3K9me2 immunofluorescence signals within the first 45 min after irradiation with 2 Gy [24]. Our observations on H3K9me3 are compatible with a transient reduction, although statistical significance was only obtained for the sample irradiated with 2 Gy and incubated for 1 h (Figure 2). Of note, the same antibody was used in our study and the work by Young et al. [24] to detect H3K9me3. We could not confirm a reduction in the amount of H3K9me2, however. In addition, we did not observe significant alterations in the amount of H3K9me1 (Figure 2).

**Figure 3:**
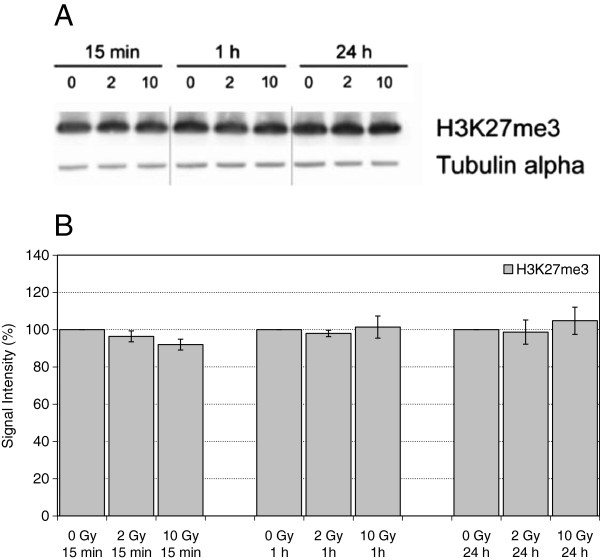
**Levels of H3K27me3 in extracts of HuKo LCLs after irradiation with 0 Gy, 2 Gy and 10 Gy and incubation for 15 min, 1 h and 24 h. A)** representative Western Blot, Tubulin alpha served as loading control. **B)** Quantitative evaluation, each normalized to unirradiated controls. Indicated are mean and standard error of the mean from 2–3 independent experiments and 1–2 blots per experiment.

H3K27me3 is a histone modification associated with gene silencing via polycomb repressive complex 2 (PRC2) (reviewed by [25]). Recruitment of members of the PRC2 complex and accumulation of H3K27me3 has been shown at DNA damage sites induced by laser irradiation or ionizing irradiation [22,26]. However, there is no evidence of global alterations in H3K27me3 levels after irradiation (Figure 3).

**Figure 4:**
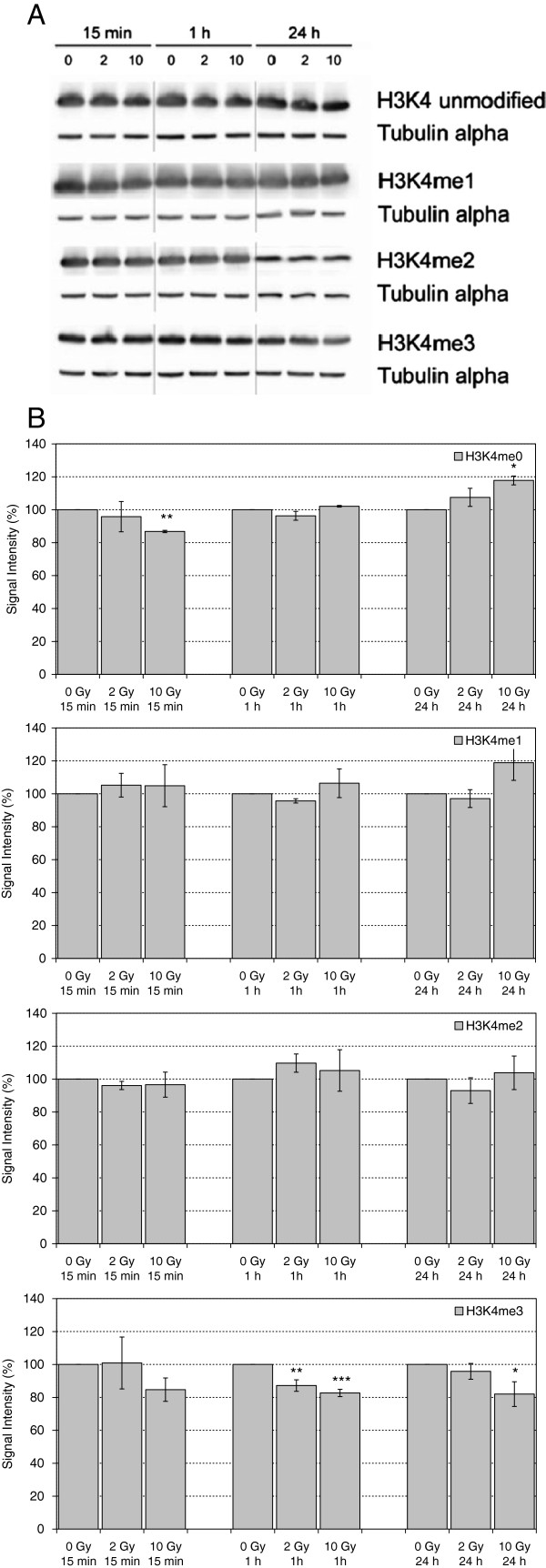
**Levels of unmodified H3K4, H3K4me1, H3K4me2 and H3K4me3 in extracts of HuKo LCLs after irradiation with 0 Gy, 2 Gy and 10 Gy and incubation for 15 min, 1 h and 24 h. A)** representative Western Blot, Tubulin alpha served as loading control. **B)** Quantitative evaluation, each normalized to unirradiated controls. Indicated are mean and standard error of the mean from 2–3 independent experiments and 1–3 blots per experiment. ** statistically significant with 0.005 > α > 0.0005, *** statistically significant with 0.0005 > α > 0.00005.

Methylation at H3K4 is associated with transcriptionally active regions, with H3K4me3 preferentially found in 5′ upstream regions of genes. Recently we demonstrated by immunofluorescence analysis a loss of H3K4me3 and H3K4me2 signals in γ-H2AX domains, which started within minutes after damage infliction by irradiation and increased over time [24]. In addition to local loss of H3K4me3, we also detected a global reduction of H3K4me3 levels by Western blot analysis in HeLa cells [24]. In the present work, a robust reduction of H3K4me3 levels was found in LCL cells under various dose and incubation time combinations (Figure 4), thus corroborating our earlier observations. Mono- and di-methylated H3K4 did not exhibit significant alterations in response to irradiation, while analysis of unmethylated H3K4 gave inconclusive results: after irradiation with 10 Gy, a transient decrease was evident after 15 min, followed by an increase after 24 h (Figure 4). Whether this pattern is causally related to the loss of H3K4me3 remains to be elucidated.

**Figure 5:**
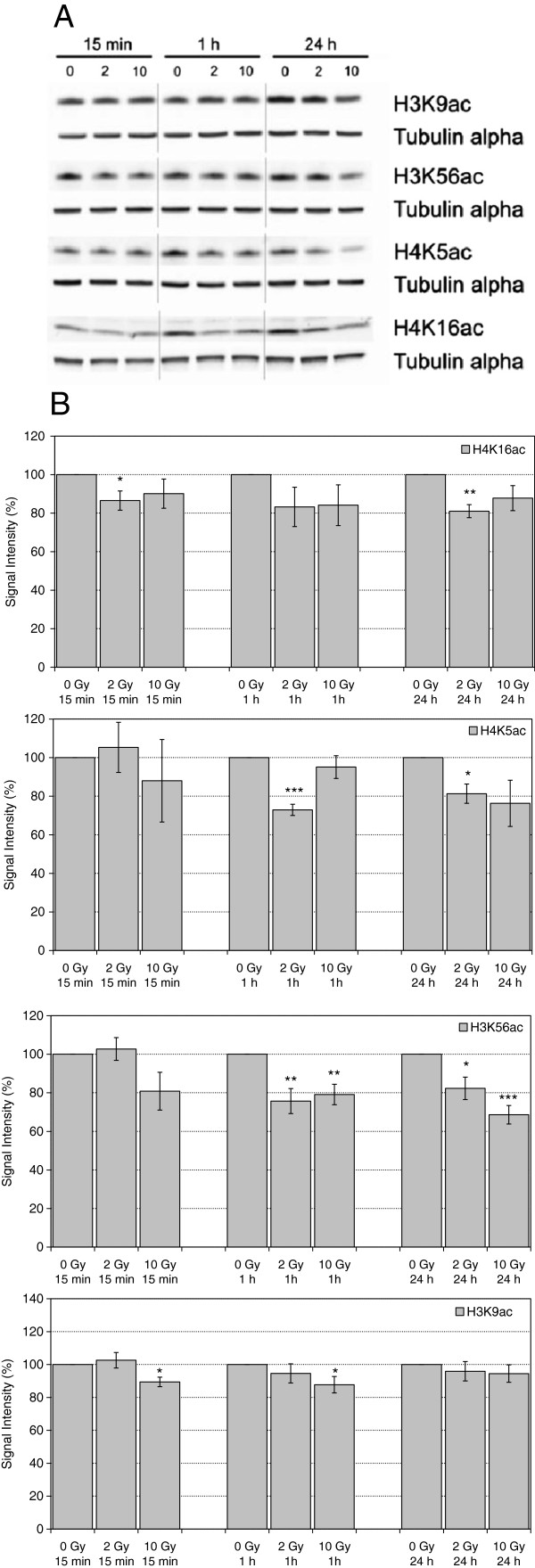
**Levels of H4K16ac, H4K5ac, H3K56ac and H3K9ac in extracts of HuKo LCLs after irradiation with 0 Gy, 2 Gy and 10 Gy and incubation for 15 min, 1 h and 24 h. A)** representative Western Blot, Tubulin alpha served as loading control. **B)** Quantitative evaluation, each normalized to unirradiated controls. Indicated are mean and standard error of the mean from 2–3 independent experiments and 1–2 blots per experiment. * statistically significant with 0.05 > α > 0.005, ** statistically significant with 0.005 > α > 0.0005, *** statistically significant with 0.0005 > α > 0.00005.

Histone acetylation is generally assumed to contribute to chromatin opening. A classic view on DNA damage response assumes that enhancement of accessibility at damage sites is necessary to warrant efficient recruitment of DNA damage response and repair proteins, and that histone acetylation, especially at the N-terminal tail of H4, plays a fundamental role in conferring enhanced accessibility (e.g., [27-30], Xu et al. 2010; reviewed by [31]. A more detailed analysis of histone acetylation patterns following DNA damage induction revealed, however, that the processes are more complex. H4K16ac is a well-characterized modification that was experimentally shown to disrupt chromatin compaction in vitro [32,33]. A biphasic regulation of H4K16ac was observed at DSB sites, with a rapid loss of acetylated H4K16 that later is followed by an increase in acetylation levels [16,34,35]. Since H4K16 acetylation diminishes 53BP1 binding to H4K20me2, rapid loss of the acetyl group after DSB induction promotes 53BP1 accumulation at the break site [16,35]. On a global level, a similar biphasic dynamics was reported by Hsiao and Mizzen [16] after Bleocin treatment, while others find an increase of H4K16ac levels already very shortly after irradiation [36,37]. In our study, we observe comparable reductions of H4K16ac levels at all time points and doses tested (Figure 5), albeit statistical significance was only obtained for samples irradiated with 2 Gy and incubated for 15 min and 24 h.

Globally detectable rapid deacetylation may occur on several N-terminal lysines of H4 [16]. We tested H4K5ac and observe a reduction in the level of this mark at several time points after irradiation, up to 24 h (Figure 5). Similar patterns were observed with H3K56ac (Figure 5), a modification the biological function of which in mammalian cells is still largely unclear. Conflicting data were reported on global H3K56ac alterations after induction of different damage types [5,34,38-40] and a detailed analysis of global H3K56ac patterns depending on radiation dose and postirradiation incubation had been lacking hitherto. To reconcile conflicting data on whether H3K56ac levels decrease or increase in γ−H2AX-decorated chromatin regions ([34,39,41,42]), a model assuming a biphasic pattern with rapid loss of H3K56ac and subsequent restoration or even overshooting accumulation was proposed, similar to the situation with H4K16ac [34,38]. However, our observations on H4K16ac, H4K5ac, and H3K56ac hint at a long-term reduction in the nuclear amount of these modifications by up to 20-30%, similar after 2 Gy and 10 Gy. Since, according to our earlier estimation, alterations that take place exclusively in the γ−H2AX domain are expected to affect genomic levels by a few percent at most, these observations suggest that not only the immediate vicinity of the DSB sites is concerned.

Another histone acetylation implicated in the DNA damage response is H3K9ac. In addition to a local loss in γH2AX-decorated radiation-induced foci [43], decreased global levels after treatment with various genotoxic agents were reported [5,44]. Our data hint at a rapid and transient decrease in H3K9ac levels, the extent of which appears to be lower than observed for the other acetylation sites tested (Figure 5).

**Figure 6:**
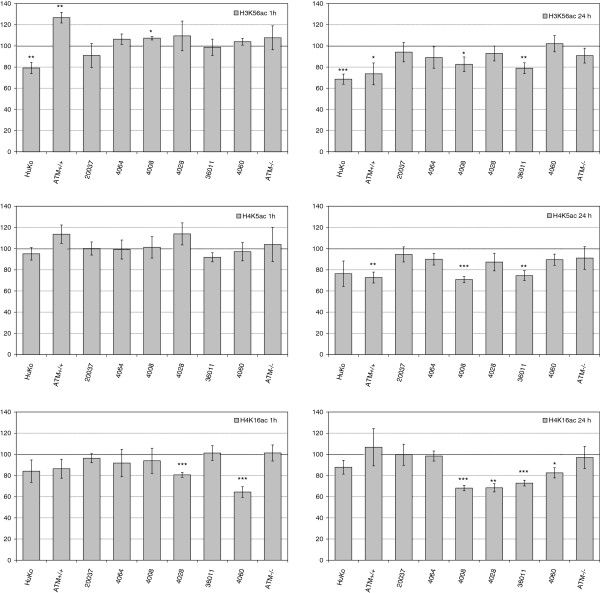
**Levels of histone modifications H3K56ac, H4K5ac and H4K16ac after irradiation with 10 Gy and incubation for 1 h and 24 h in extracts from radioresistant LUCY LCLs 20037 and 4064, radiosensitive LUCY LCLS 4008, 40028, 36011, and 4060, as well as LCLs from ATM proficient and ATM deficient individuals.** Data from HuKo LCLs are shown for comparison. Indicated are means and standard errors of the mean from 2 independent experiments and 2–8 blots per data point. * statistically significant with 0.05 > α > 0.005, ** statistically significant with 0.005 > α > 0.0005, *** statistically significant with 0.0005 > α > 0.00005.

To further elucidate the reason for the high preponderance of conflicting reports on alterations in histone PTM patterns after DSB induction, and to determine whether the observed differences may be associated with different radiosensitivity, we expanded our analysis to a panel of LCL lines established from young lung cancer patients (LUCY cohort; [45]). In prior work radiation sensitivity of these LCLs was established using viability assays (Gürtler et al. 2010). We consider the lines 20037 and 4064 as relatively resistant, with viability levels similar to the ATM-proficient cell line GM03323, which was included as a control. In contrast, the sensitive lines 4008, 4028, 36011, and 4060 exhibit survival levels comparable to the ATM-deficient cell line GM03189. As compared to the resistant lines, all sensitive lines exhibit higher levels of residual γ−H2AX foci at 24 h after irradiation with 10 Gy, hinting at compromised DSB repair in these lines (Table 3). We investigated H3K56ac, H4K5ac and H4K16ac in these cells lines as these PTMs yielded rather robust alterations in the screen with HuKo LCLs. Histone acetylation levels were investigated 1 h and 24 h after irradiation with 10 Gy (Figure 6). We note a prominent inter-individual variability of quality and quantity of alterations in the acetyl marks tested, which in general seems not to be related to radiation sensitivity, with the exception of residual H4K16 hypoacetylation at 24 h after irradiation. In all four sensitive cell lines established from lung cancer patients, the extent of hypoacetylation at H4K16 was stronger than in any of the radioresistant lines. With reductions in the range of 20-30%, it is possible that hypoacetylation involves not only the γ-H2AX domains, but additional genomic regions. Further experiments are necessary to clarify this question.

**Table 3:** Residual γ-H2AX foci 24 h after γ-irradiation with 10 Gy

**Cell line**	**Residual γ-H2AX foci (compared to unirradiated control)**
ATM +	119% ± 5%
20037	101% ± 5%
4064	99% ± 8%
4008	172% ± 4%
4028	169% ± 9%
36011	167% ± 9%
4060	177% ± 11%
ATM -	141% ± 4%

Shown the mean and standard deviation of two independent experiments.

While our sample size is too small to allow final conclusions, a correlation between prolonged H4K16 hypoacetylation and radiosensitivity or reduced DSB repair would be intriguing. It is possible that reduced repair retards re-establishment of chromatin structure, therefore protracting hypoacetylation. On the other hand, it is also conceivable that reduced repair is causally linked to prolonged hypoacetylation of H4K16: recent work showed that hypoacetylation at H4K16 results in enhanced binding of 53BP1 and thus reduced binding of Brca1. The presence of 53BP1 blocks resection and thus facilitates DSB repair via non-homologous end joining, while the presence of Brca1 would promote resection and repair via homologous recombination [16,34,35]. The outcome of prolonged H4K16 hypoacetylation thus may resemble the phenotype of Brca1 deficiency or TIP60 deficiency, i.e. reduced repair via homologous recombination, more aberration formation, and increased sensitivity to PARP inhibitors [16,34,35]. Others have proposed that H4K16 hypoacetylation leads to a general reduction of DSB repair [46-48]. In addition, H4K16 hypoacetylation may lead to transcriptional deregulation [49,50] and thus affect radiosensitivity. H4K16 hypoacetylation has been implicated as a hallmark of cancer [51]. Both class I and class III histone deacetylases (HDACs) can deacetylate H4K16ac [52]. Inhibitors of HDAC are increasingly considered as cancer therapeuticals that have a potential to radiosensitize tumour cells [53]. Our observations suggest, however, that in some situations the inhibition of H4K16 hypoacetylation may render cells more resistant towards the effects or irradiation. Clearly, more research is needed to clarify the relation between radiation response and histone hypoacetylation.

## Conclusions

By screening a variety of histone PTMs for radiation-induced alterations in a normal human LCL, we observed little variation in histone methylation marks, except for tri-methylation of H3K4 the levels of which were reduced after irradiation. In contrast, consistent alterations in all acetylation marks tested suggest a rather long-lasting, globally detectable hypoacetylation. In a panel of LCLs established from lung cancer patients, we observed, however, a high degree of variability with regard to radiation-induced alterations in histone acetylation. Of special interest, long-term hypoacetylation of H4K16 was strongest in cell lines exhibiting increased radiosensitivity and enhanced levels of residual γ-H2AX foci. This observation may have implications for the use of HDAC inhibitors in radiation oncology.

## Abbreviations

BSA: Bovine serum albumin; DSB: Double-strand break; EBS: Epsein-Barr-Virus; IRIF: Ionizing radiation-induced foci; LCL: Lymphoblastoid cell line; LUCY: Lung cancer in the young; PBS: Phosphate-buffered saline; PRC2: Polycomb repressive complex 2; PTM: Post-translational modification.

## Competing interests

The authors declare no competing interests.

## Author’s information

Belinda Maroschik is née Mazurek.

## Authors’ contributions

AAF and BM designed screening study. BM performed analysis of histone modifications, AG cultivated LCLs and, together with AK, performed radiosensitivity analyses, AK and UR performed γH2AX foci analysis. All authors designed characterization of LUCY cell lines. MG and SH provided materials. AAF wrote the manuscript with contributions of BM and SM. All authors read and approved the final manuscript.
